# Integrin Activation Through the Hematopoietic Adapter Molecule ADAP Regulates Dendritic Development of Hippocampal Neurons

**DOI:** 10.3389/fnmol.2016.00091

**Published:** 2016-09-30

**Authors:** Marlen Thiere, Stefanie Kliche, Bettina Müller, Jan Teuber, Isabell Nold, Oliver Stork

**Affiliations:** ^1^Department of Genetics and Molecular Neurobiology, Institute of Biology, Otto-von-Guericke-UniversityMagdeburg, Germany; ^2^Institute of Molecular and Clinical Immunology, Medical Faculty, Otto-von-Guericke-UniversityMagdeburg, Germany; ^3^Center for Behavioral Brain SciencesMagdeburg, Germany

**Keywords:** integrin, adaptor proteins, neurite outgrowth, MAP2, hippocampus, primary cell culture, mouse

## Abstract

Integrin-mediated cell adhesion and signaling is of critical importance for neuronal differentiation. Recent evidence suggests that an “inside-out” activation of β1-integrin, similar to that observed in hematopoietic cells, contributes to the growth and branching of dendrites. In this study, we investigated the role of the hematopoietic adaptor protein adhesion and degranulation promoting adapter protein (ADAP) in these processes. We demonstrate the expression of ADAP in the developing and adult nervous hippocampus, and in outgrowing dendrites of primary hippocampal neurons. We further show that ADAP occurs in a complex with another adaptor protein signal-transducing kinase-associated phosphoprotein-homolog (SKAP-HOM), with the Rap1 effector protein RAPL and the Hippo kinase macrophage-stimulating 1 (MST1), resembling an ADAP/SKAP module that has been previously described in T-cells and is critically involved in “inside-out” activation of integrins. Knock down of ADAP resulted in reduced expression of activated β1-integrin on dendrites. It furthermore reduced the differentiation of developing neurons, as indicated by reduced dendrite growth and decreased expression of the dendritic marker microtubule-associated protein 2 (MAP2). Our data suggest that an ADAP-dependent integrin-activation similar to that described in hematopoietic cells contributes to the differentiation of neuronal cells.

## Introduction

The nervous system and the immune system share many mechanisms concerning the recognition of cells and extracellular matrix components, as well as the intracellular signaling induced by these events. The adhesion and degranulation promoting adapter protein (ADAP) may be a common regulatory factor in these processes. ADAP is expressed in various hematopoietic cells including T-cells, platelets, mast cells, dendritic cells, natural killer cells, granulocytes, monocytes, macrophages (Wang and Rudd, 2008; Witte et al., 2012) and microglia (Engelmann et al., 2015), but public databases suggest that ADAP may also be expressed in neuronal cells during development and adulthood^1^.

ADAP protein occurs in two isoforms with molecular weights of 120 kDa and 130 kDa, without discernable enzymatic or transcriptional activity (Wang and Rudd, 2008). It contains a proline-rich region, several tyrosine-based signaling motifs, two helical SH3 domains, and an Ena/VASP binding motif to mediate protein-protein and protein-lipids interactions (Peterson, 2003; Wang and Rudd, 2008; Witte et al., 2012; Engelmann et al., 2015). It serves as a hub for the association of additional adaptor proteins, Ena/VASP proteins and kinases in T-cells, thereby facilitating T-cell activation, differentiation and adhesion (Peterson, 2003; Zhang and Wang, 2012; Witte et al., 2012). ADAP deficient T-cells show reduced T-cell receptor (TCR)-mediated differentiation and proliferation and an attenuated up-regulation of the T-cell activation markers CD69, CD25 and CD54 as well as release of interleukin-2 and interferon γ (Peterson et al., 2001; Medeiros et al., 2007; Wang et al., 2007; Burbach et al., 2008; Srivastava et al., 2010). In addition, loss of ADAP attenuates TCR- and chemokine-mediated integrin activation required for T-cell adhesion, interaction with antigen-presenting cells and migration *in vitro* and *in vivo* (Peterson et al., 2001; Wang et al., 2007; Burbach et al., 2011; Kliche et al., 2012; Mitchell et al., 2013). However, the potential role of ADAP in integrin activation during neuronal differentiation has not been studied so far.

Neurons express various β1- and β3-integrins (Wu and Reddy, 2012) that interact with the rich extracellular matrix of the nervous system (e.g., fibronectin, laminin, or collagens) and with diffusible factors that serve as guidance cues mediating migration and neurite growth (e.g., netrins, semaphorins and ephrins; Myers et al., 2011). Beta-integrins are expressed during dendritic differentiation (Schmid and Anton, 2003; Rehberg et al., 2014) and provide sites of adhesion and signals for the dynamic rearrangement of cytoskeletal elements during dendrite development. Stimulation of integrins with laminin or semaphorin 7A enhances the growth and restructuring of dendrites in cortical neurons in culture (Moresco et al., 2005), whereas integrin blockage leads to retraction of dendrites of retinal ganglion cells *in vivo* (Marrs et al., 2006). Hippocampal neurons also require β1-integrins for dendritic differentiation both in culture and *in vivo* (Schlomann et al., 2009; Warren et al., 2012; Rehberg et al., 2014). While classically it has been considered that integrins in neurons are expressed in a pre-activated state and mostly mediate signaling from the extracellular matrix and diffusible factors (“outside-in”), recent evidence has demonstrated the importance of controlled integrin trafficking and “inside-out” activation during neurite development. Specifically, increased expression of activated β1-integrin on the dendritic surface has been reported following stimulation of hippocampal neurons with semaphorin 3A (Schlomann et al., 2009; Rehberg et al., 2014).

These processes bear striking resemblance to the ADAP-dependent inside-out activation of integrins in T-cells, where upon stimulation of the TCR or chemokine receptors, αLβ2 and α4β1 integrins are activated to bind to their respective ligands. Consequently an increased proportion of integrins is induced to a high-affinity conformation on the cell surface (affinity modulation), followed by integrin clustering and association with the actin cytoskeleton (avidity regulation; Abram and Lowell, 2009; Hogg et al., 2011).

ADAP in T-cells is associated with SKAP55 to regulate the affinity/avidity modulation of integrin function via the assembly of two complexes, ADAP/SKAP55/RAPL/MST1 and ADAP/SKAP55/RIAM/MST/Kindlin-3/Talin, which are associated with the alpha or beta chain of the integrin αLβ2, respectively (Kliche et al., 2012). Three components of the ADAP associated molecular complex in T-cells, Talin, Kindlin-1 (an isoform of Kindlin-3) and the Rap1 effector protein RIAM have previously also been found to regulate β1- and β3-integrin function in neurons (Dent et al., 2011; Myers et al., 2011; Tan et al., 2012).

Based on these observations and its prominent expression in the nervous system, we hypothesized that ADAP may be involved in the activation of integrins during neuronal differentiation. We examined the expression of ADAP during dendritogenesis of cultivated hippocampal neurons and investigated the effect of ADAP knock down on neuronal differentiation and underlying mechanisms. Our data suggest that ADAP occurs in developing neurons in association with signal-transducing kinase-associated phosphoprotein-homolog (SKAP-HOM; homolog of SKAP55), RAPL and MST1, and stimulates β1 integrin activation as well as dendritic growth in these cells.

## Materials and Methods

### Mice

C57BL/6 (M&B Taconic, Berlin) mice were bred and maintained under specific pathogen-free conditions at the Otto-von-Guericke University, Magdeburg, Germany. Animal maintenance and tissue collection were done according to the guidelines of the State of Saxony-Anhalt, Germany and approved by the Landesverwaltungsamt Sachsen-Anhalt.

### Cell Culture

HEK-293T cells (supplied by *Deutsche Stammsammlung von Mikroorganismen und Zellkulturen GmbH; DSMZ* Braunschweig, Germany) were used for testing plasmid constructs. Transfection was done with Lipofectamine^®^ 2000 (Thermo Scientific) according to the manufacturer’s protocol. For Western Blotting, cells were lyzed 48 h after transfection. PC-12 cells were cultured in RPMI medium containing 10% horse serum (v/v), 5% fetal bovine serum (v/v) and 1% L-Glutamine (v/v; all Thermo Scientific). Differentiation was induced with NGF (50 ng/μl; Sigma-Aldrich) under reduced serum condition [RPMI medium containing 0, 2% horse serum (v/v) and 1% L-glutamine (v/v)]. Splenic CD3^+^ T-cells from mice were purified using T-cell isolation kit and AutoMacs magnetic separation system (Miltenyi Biotec).

### Primary Hippocampal Culture

Dissociated primary hippocampal cultures were prepared using the Neural Tissue dissociation Kit (P) from Milteny Biotec according to manufacturer’s protocol. Briefly, hippocampi from embryonic day 18 (E18) mice were dissected, dissociated in papain-enzyme mix and incubated under rotation at 37°-C for 15 min in Hanks balanced salt solution (HBBS, Thermo Scientific). Dissociated cells in DMEM were plated at a density of 40,000–80,000 cells/cm^2^ (for transfection) or 200,000 cells/cm^2^ (for immunocytochemistry) on poly-D-lysine-coated (Sigma-Aldrich) coverslips. Four hours after plating, DMEM containing 10% FBS (v/v), 2 mM L-GlutaMAX, was changed to Neurobasal^®^ medium containing 2% B27- supplement (v/v; all Thermo Scientific), 0.5 mM L-GlutaMAX. After 2 days *in vitro* (DIV), cells were treated with 10 μM AraC (Sigma-Aldrich) to inhibit Glia proliferation. On DIV7 media was changed to neurobasal medium containing 2% B27 (v/v) supplement without GlutaMAX. Neuronal cultures were transfected at DIV7 using Lipofectamine^®^ 2000 (Life Technologies), according to the manufacturer’s protocol (see above). After transfection, coverslips were cultured for two additional days in neurobasal medium containing 2% B27 (v/v). The developmental stages of the transfected neurons were carefully monitored (Kaech and Banker, 2006) and their viability was evaluated according to the smoothness and regularity in shape of somata and the uniformity in diameter and smoothness of neurites (Xiang et al., 1996). We also controlled for phase bright somata and granule accumulation (Yang et al., 2010). Transfection did not result in a change of these parameters, or in the occurrence of fragmented neurites or rough, condensed and irregularly shaped somata in any experimental group.

### Constructs

The expression vector pll.3.7 (Rubinson et al., 2003) was obtained from Addgene and used for cloning of ADAP shRNA targeting oligonucleotide. ADAP shRNA sequences targeting both isoforms of the mouse ADAP mRNA (NM_011815.5/NM_001278269.1) were designed using the *shRNA retriever* online tool^2^. Hairpin oligonucleotides with the loop sequence TTCAAGAGA were cloned into pll3.7 downstream of its U6 promoter, using Hpa1 and Xho1 restriction sites. This construct was co-transfected with a murine ADAP overexpressing construct in HEK-293T cells to test the efficiency to knock down mouse ADAP mRNA (Figure 2C). Ultimately, an shRNA construct expressing the fragment GCCAGGATTCTCAAAGGTAGC and targeting nucleotides 572–593 was chosen for further experiments. As controls we used both, a nonsense construct [pll3.7-shrandom (5^′^ TCGTCATGACGTGCATAGG 3^′^)] and a pll3.7 empty backbone in all experiments. These controls did not differ in any of the parameters analyzed and therefore were averaged for statistical analysis and data presentation. Moreover, a cDNA clone of Flag-tagged human ADAP (Musci et al., 1997), insensitive to these knock-down constructs was used for reconstitution of ADAP expression. Control experiments revealed that an expression of ADAP from this vector in the absence of a knock-down constructs frequently induced aberrant morphology, in particular axon swellings, that were never observed in the other experimental conditions including the rescue groups. Therefore, ADAP overexpression was not further considered in our experiments. In control, knock down and reconstitution vectors, enhanced green fluorescence protein was independently expressed under a cytomegaly virus promoter from the same construct to visualize transfected cells. To confirm antibody specificity we furthermore inserted murine His-tagged ADAP into vector pCMS4 for heterologous expression in HEK293T cells. For Luciferase reporter assays, the plasmids pGL4.32 [luc2P/NF-kB-RE/Hygro] and pRL-TK were obtained from Promega.

### Immunocytochemistry

Immunocytochemistry and neurite growth analysis were done using a modified protocol from Rehberg et al. (2014). For immunocytochemistry primary neurons were fixed with 4% paraformaldehyde and 4% sucrose in 0.1 M PBS, pH 7.4. Cells were permeabilized with PBS containing 0.3% Triton X-100 and unspecific binding was blocked with 10% BSA in PBS, followed by primary antibody incubation in blocking solution at room temperature for 1–2 h. Cells were washed in PBS, incubated for 1 h at room temperature with suitable Alexa conjugated secondary antibodies in 2.5% bovine serum albumin in PBS [donkey anti-mouse Alexa 647 (Thermo Scientific), donkey anti-sheep Cy3 (Dianova), donkey anti-rat Cy3 (Dianova)]. Cells were again washed with PBS, embedded with Immu-Mount (Thermo Scientific) and examined using Leica DMIR2 confocal and Leica DMI6000 epifluorescence microscopes.

### Morphological Assessment of Transfected Neurons

Transfected primary neurons were fixed with 4% paraformaldehyde and 4% sucrose in 0.1 M PBS, pH 7.4. Dendrites of GFP-filled neurons were identified according to their morphological features (Kaech and Banker, 2006) and MAP2 counterstaining. Dendritic arborization was then evaluated according to the method of Sholl (1953), using a DMI6000 microscope and QWin software (Leica Microsystems).

### Analysis of Activated β1-integrin and Total β1-integrin

Integrin activation was examined using an antibody for the high affinity conformation of CD29 (Ab 9EG7; BD Bioscience) according to a modified protocol from Tan et al. (2012) and Rehberg et al. (2014). The antibody was added to a final dilution of 1:50 to the culture medium and incubated for 15 min at 37°C. Cells were then washed with warm culture medium and fixed with 4% PFA, 4% sucrose in PBS for 30 min. Cells were permeabilized with PBS containing 0.3% Triton-X and counterstained with an antibody against MAP2 (1:1000; Millipore). Total β1-integrin was stained with an antibody against β1-integrin (1:500; Abcam). After washing in PBS, Alexa 647-coupled anti-mouse (Thermo Scientific); Alexa 555-coupled anti-rabbit (Thermo Scientific) and Cy3-coupled anti-rat (Dianova) secondary antibodies were applied for 1 h at room temperature. Cells were washed in PBS and mounted using ImmuMount^TM^. Each 10 GFP-labeled cells per condition and experiment were randomly selected under the DMI6000 light microscope and the immunofluorescence signal was quantified using the inbuilt LAS AF software under identical light intensity and exposure settings between cells. Each cell was tracked as a stack of 30 images with a width of 0.2 μm per image. Blind deconvolution was performed, dendrites and soma of each neuron were traced and fluorescence intensity was analyzed with the histogram tool of the LAS AF software. Labeling intensity was expressed in relation to the surface area of the respective compartment, as visualized by the EGFP expressed from the transfected construct. Morphological parameters and MAP2 counterstaining were used to differentiate dendritic, somatic and axonal compartments.

### Quantitative PCR

RNA isolation and first strand synthesis were done as previously described (Albrecht et al., 2013; Rehberg et al., 2014). In brief, RNA was isolated from mouse primary hippocampal neurons on DIV3, 7, 14 and 21 using Cells-to-cDNA II^TM^-Cell Lysis Buffer (Ambion^®^). cDNA was generated with M-MLV reverse transcriptase Omniscript (Qiagen) using oligo-dt primers and random decamer primers. Quantitative PCR was done on a StepOnePlus real-time PCR System using TaqMan reagents and TAM-labeled predesigned expression assays for ADAP (Mm00803629_m1), p65 (Mm00501346_m1) or c-Rel (Mm01239661_m1; all Thermo Scientific). Initial deuridination and denaturation (2 min 50°C, 10 min 95°C) were followed by 40 cycles of 15 s 95°C, 1 min 60°C and expression values were calculated in relation to those obtained with the VIC-labeled housekeeping gene assay for GAPDH (4352923E) in the same wells.

### Western Blotting

Western blotting was done as previously described (Rehberg et al., 2014). Briefly, hippocampi and cultured cells were lyzed in laurylmaltosid/NP40 lysis buffer, (1% lauryl maltoside N-dodycyl-D-maltoside (Merck), 1% NP-40 (Sigma-Aldrich), 1 mM Na-orthovanadate, 1 mM PMSF, 50 mM Tris-HCl, pH7.4, 10 mM NaF, 10 mM EDTA, and 160 mM NaCl) incubated on ice for 20 min and centrifuged at 16000× g for 30 min. The protein concentration of the postnuclear supernatant was determined using the Roti-Nanoquant reagent (Roth) according to the manufacturer’s instructions. Cell lysates or precipitates were separated by SDS-PAGE and transferred to PVDF or nitrocellulose membranes (Immobilon FL; Millipore). The following antibodies were used sheep anti-ADAP [kindly provided by Gary Koretzky University of Pennsylvania; (Musci et al., 1997)] mouse anti-α-tubulin (Sigma-Aldrich); mouse anti-ADAP mAb (BD Bioscience), rabbit anti-ADAP (EPR2547Y; Abcam), as well as rat mAbs against Riam and RAPL (Horn et al., 2009; Kliche et al., 2012), MST1 (BD Bioscience) and SKAP-HOM (Marie-Cardine et al., 1998). Membranes were then incubated with horseradish peroxidase-conjugated secondary antibodies (Dianova) and signals were detected with a Luminol^TM^ detection system (Roth) exposing to X-ray films (Amersham).

### Immunoprecipitation

Immunoprecipitation (IP) was performed to identify protein-protein interaction. Total cell lysate (500 μg) were supplemented with 30 μg BSA to reduce non-specific binding, the ADAP sheep serum (10 μl) and 30 μl Protein A-agarose (Santa Cruz) for 2 h at 4°C. After washing the beads with laurylmaltosid/NP40 lysis buffer, precipitates were analyzed by Western Blotting as described above.

#### In-Cell Western

MAP2-immunoreactivity in transfected cell cultures was quantified with an In-Cell Western^TM^ assay. Dissociated neurons were transfected with Lipofectamine^®^ 2000 (Thermo Scientific) on DIV7 and fixed on DIV9 with 4% PFA/4% sucrose followed by permeabilization with PBS containing 0.1% Triton-X at room temperature. Primary anti-MAP2 antibody (1:200, Millipore) was diluted in Odyssey^®^-blocking buffer (LI-COR^®^) and plates were incubated at 4°C over night. After washing in PBS, 0.1% Tween (5 × 5 min), a secondary antibody (1:1000; IRDye^®^ 800CW goat anti-mouse) was applied together with CellTag^TM^ 700 stain (1:500; LI-COR^®^) for 1 h at room temperature. After washing in PBS, 0.1% Tween (5 × 5 min at room temperature) fluorescence intensity signals were analyzed with the Odyssey^®^-Infrared Imager (LI-COR^®^) and MAP2 signals were normalized to the total cell number as detected with the CellTag^TM^ 700 stain.

#### Luciferase Assays

To detect NF-κB-activity in stimulated primary hippocampal neurons vs. unstimulated neurons, a Dual Glow^®^ Luciferase assay system (Promega) and pGL4.32[luc2P/NF-κB-RE/Hygro] reporter were used. Neurons were transfected on DIV7 with NF-κB-Luc reporter and a Renilla-Luciferase control vector (pRL-TK; Schultz et al., 2006; Mikenberg et al., 2007). TNFα (100 ng/ml) and Insulin (10 μg/ml; both Sigma-Aldrich) stimulation were done on DIV9 for 90 min before measurement commenced. Dual-Luciferase^®^ reporter assay was performed according to the manufacturer’s protocol. Briefly, after washing with PBS cell were lyzed with 1× passive lysis buffer (5× PLB; Promega) for 15 min at room temperature. 1× luciferase assay buffer II (LARII) was added and after 10 min firefly luminescence was measured using a Luminescence spectrophotometer (Tecan Infinite^®^ M200). The same volume of 1× Stop & Glow^®^ was added and after 10 min, Renilla luminescence was measured. Reporter activity was calculated as the ratio of experimental reporter pGL4.32 [luc2P/NF-kB-RE/Hygro] luminescence to control reporter pRL-TK luminescence and normalized to control pLL3.7 controls.

#### Statistics

Statistical analysis was performed with one-way ANOVA followed by Fischer’s protected least significant difference (PLSD) test. Student’s *t*-test was used for direct pairwise comparisons. A *p*-value of *p* < 0.05 was considered to be significant.

## Results

### ADAP is Expressed in Neuronal Cells

**Figure 1:**
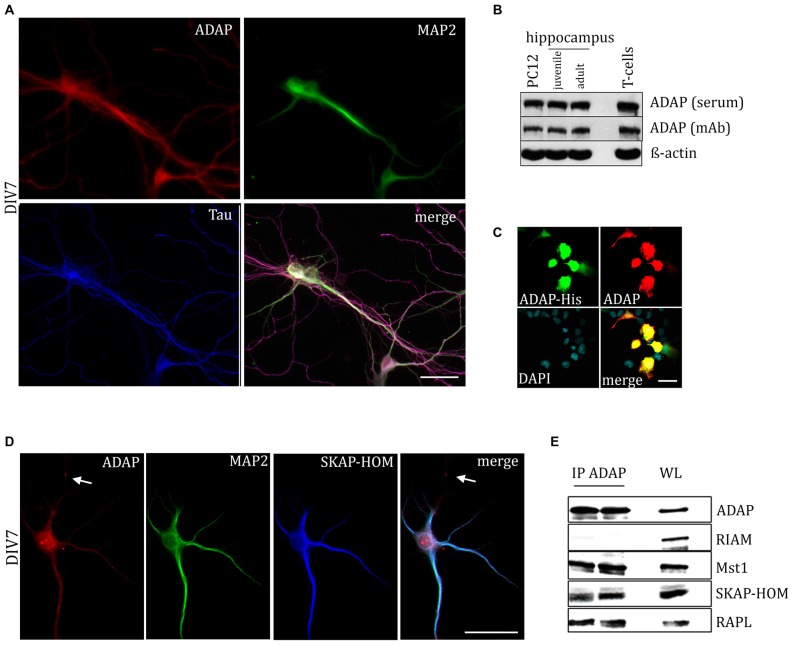
**Expression of adhesion and degranulation promoting adapter protein (ADAP) in hippocampal neurons. (A)** ADAP expression can be detected in the somata of primary hippocampal neurons and along their MAP2-positive dendritc and MAP2-negative/Tau-positive axonal structures. Scale bar, 100 μm. **(B)** Immunoblot analysis with two different ADAP antibodies confirms the expression in juvenile (postnatal day 9) and adult (postnatal day 90) hippocampus, as well as in neurally differentiated PC12 cells (after 4 days of NGF treatment). ADAP expression in murine CD3^+^ T-cells is shown for comparison.** (C)** The specificity of immunocytochemical ADAP labeling is demonstrated in HEK-293T cells, which do not express endogenous ADAP. Positive signals are strictly limited to those cells that have been transfected with His-tagged ADAP, whereas non-transfected cells marked by DAPI staining alone are negative for ADAP immunoreactivity. Scale bar, 100 μm. **(D)** In 7 days *in vitro* (DIV7) hippocampal neurons, SKAP-HOM is distributed in soma and proximal dendrites along with ADAP, but not in a MAP2-negative neurite (arrow). Scale bar, 100 μm. **(E)** Immunoprecipitation (IP) of hippocampal lysate identifies SKAP-HOM, RAPL and MST1, but not RIAM as binding partners of ADAP in neuronal tissue. WL, whole lysate.

ADAP expression was tested in primary hippocampal cultures, as well as in hippocampal tissue during development and adulthood. Immunocytochemical staining revealed ADAP expression in somata, dendrites and axons of primary neurons (Figures 1A,D, 4), as various stages of neuronal differentiation, including DIV3 (Figure 4A), DIV7 (Figures 1A,D), DIV10 (Figure 4B), as well as DIV14, DIV18 and DIV21 (data not shown).

We further examined the expression of ADAP in different compartments of the neuronal cell during development. Double immunocytochemistry reveals a high degree of overlap with the dendritic marker MAP2. The co-localization is pronounced during early neuronal differentiation (DIV3), when MAP2 labels both outgrowing axons and dendrites. At this time point, ADAP can be found along the core neurite microtubules and the microtubule network of growth cones and growth tips and MAP2-negative ADAP-positive filaments are rarely observed (Figure 4A). However, at later stages of development ADAP-positive axons without MAP2 labeling are frequently observed in addition to the generally double-labeled dendritic structures (Figures 1A,D).

Western blot analysis further confirmed the expression of ADAP in the developing and adult hippocampus *in vivo*, with two different antibodies that detected a band of approx. 120 kDa corresponding to the ADAP signal obtained in naive T-cells. ADAP was also found in neural differentiated PC12 cells (Figure 1B).

The specificity of immunocytochemical ADAP labeling was confirmed using heterologous expression of ADAP-His tagged protein in HEK-293T cells, which are devoid of endogenous ADAP expression. Only cells with detectable signal against the His-Tag (1:500; Santa Cruz) displayed immunoreactivity for ADAP antibodies (Figure 1C).

### ADAP Occurs in an Adaptor Protein/Signaling Complex in Neural Cells

In T-cells ADAP exists in complex with SKAP55, RAPL or RIAM, and the Hippo kinase MST1. This complex is known to mediate TCR-induced inside-out activation of integrins. Indeed, staining of hippocampal neurons for SKAP-HOM resulted in a distributed labeling of somata and dendrites, similar to ADAP (Figure 1D). Moreover, using anti-ADAP antibodies, we were able to co-precipitate the SKAP55 homolog SKAP-HOM, as well as RAPL and MST1, but not RIAM from hippocampal tissue (Figure 1E).

### ADAP is Required for Expression of Activated β1-integrin on Developing Dendrites

**Figure 2:**
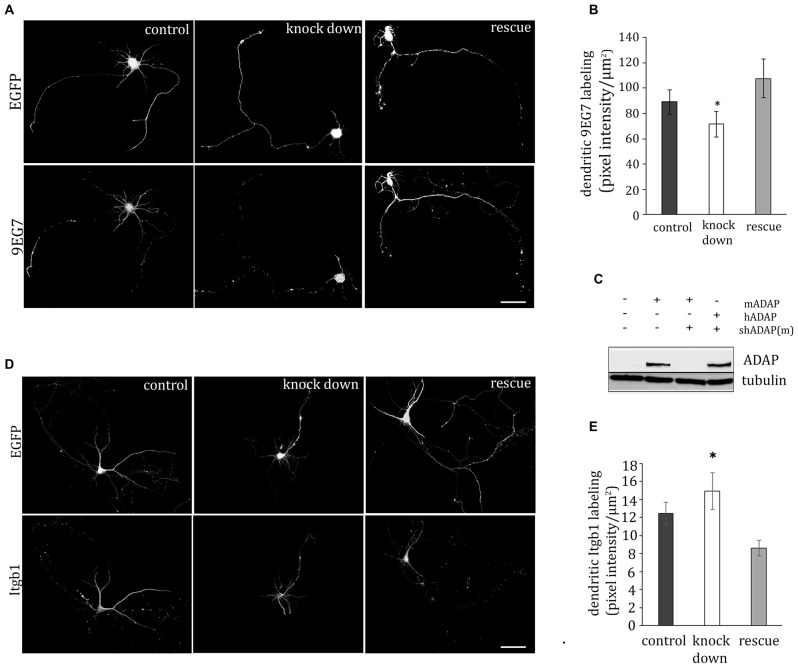
**ADAP complex and integrin activation. (A)** ADAP is involved in the dendritic expression of activated β1-integrin in hippocampal neurons (DIV9). Antibody 9EG7 detects activated β1-integrins on the soma and neurites; reduced labeling intensity is evident in cells with shRNA-mediated knock down of ADAP and recovery to control levels is seen upon re-expression of a knock-down resistant ADAP clone. Scale bar, 100 μm** (B)** Quantitative analysis of labeling intensity along dendrites confirms the reduction of 9EG7 labeling upon ADAP knock down as well as its rescue (*n* = 24–30 per condition). **(C)** Effective knock down of murine ADAP and its rescue through re-expression of the shRNA-resistant human form (hADAP) in HEK-293T cells.** (D,E)** In contrast to the activated form, total β1-integrin was increased on the dendrites following ADAP knock down, indicating that the observed reduction in dendritic labeling with antibody 9EG7 is related to reduced β1-integrin activation by conformational change rather than changes in expression level. Data are Mean ±SEM. *p < 0.01.

In T-cells the ADAP/SKAP55 module is critically involved in the inside-out activation of integrins. We therefore analyzed the expression of activated β1-integrin in somata, dendrites and axons of acutely transfected primary neurons. In fact, in dendrites ADAP knock down resulted in a decreased labeling with the activity dependent β1-integrin antibody 9EG7. Labeling was recovered to control levels, when a shRNA-resistant form of human ADAP was co-expressed (*F*_(2,145)_ = 3.208, *p* < 0.05; control vs. knock down *p* < 0.05, Fischer’s PLSD; Figures 2A,B). By contrast, in the somata (control 303.305 ± 24.39, knock down 202.32 ± 40.75, rescue 284.86 ± 79.41; *F*_(2,70)_ = 2.026 *p* > 0.05) and axons (control 75.715 ± 24.29, knock down 94.8 ± 47.15, rescue 61.644 ± 15, 21; *F*_(2,65)_ = 0.339 *p* > 0.05) no significant changes were found, although a general trend towards reduction of activated β1-integrin was apparent after ADAP knock down compared to the control.

Next, we investigated the expression of total β1-integrin on dendrites, axons and somata, using an activation stage-independent antibody. On the dendrites of ADAP knock-down cells (Figures 2D,E) we found an increase in β1-integrin intensity compared to rescue and control condition (*F*_(2,204)_ = 10.232, *p* ≤ 0.005; knock down vs. rescue *p* ≤ 0.005; knock down vs. control *p* ≤ 0.05; rescue vs. control *p* ≤ 0.005). At the same time, the somata of these cells showed a reduction in β1-integrin intensity (control 62.33 ± 1.9, knock down 38.78 ± 3.5, rescue 69.68 ± 5.7; *F*_(2,103)_ = 6.307, *p* ≤ 0.005; knock down vs. rescue *p* ≤ 0.005; knock down vs. control *p* ≤ 0.005). No significant change in β1-integrin labeling was found in the axonal compartment (control 9.27 ± 1.21, knock down 11.09 ± 1.28 rescue 8.35 ± 1.31; *F*_(2,110)_ = 1.153 *p* ≥ 0.05).

### ADAP Promotes Neurite Outgrowth

**Figure 3:**
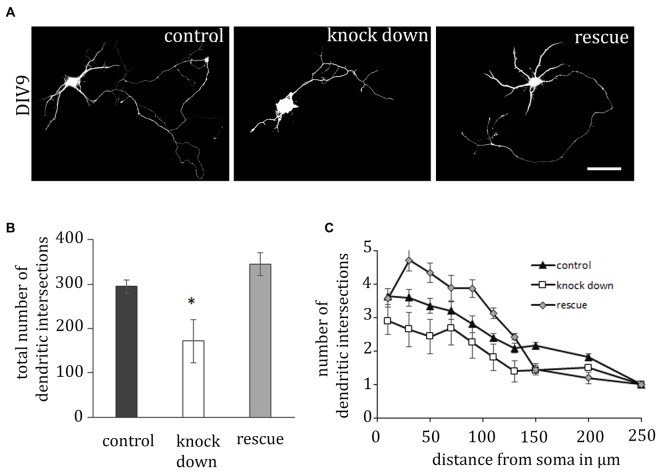
**ADAP is required for dendritic outgrowth. (A)** The complexity of dendritic structures is reduced upon ADAP knock down and recovered upon expression of the rescue construct. Dendrite formation was analyzed in primary hippocampal neurons based on the reconstruction of transfected cells via the EGFP signal. Scale bar, 100 μm. **(B)** Quantification of dendrite branches using Sholl’s method revealed a significant reduction in the total number of dendritic intersections (*n* = 25–29 per condition). **(C)** Reduction of intersections is evident in knock-down cells along the entire dendritic arbor, whereas the rescue construct enhances growth over the first 150 μm. Data are Mean ± SEM. *p < 0.01.

Activation of β1-integrin is critical for dendritic development in neuronal cells (Schlomann et al., 2009; Rehberg et al., 2014); therefore we next examined the potential effect of ADAP knock down on dendrite formation in primary hippocampal neurons (Figure 3). Here we observed that shRNA-mediated ADAP knock-down induces a significant reduction of neurite growth, which can be recovered by co-expression of an shRNA-resistant ADAP expression construct. Quantification of neurites with the Sholl method demonstrated a significant reduction in the number of dendritic intersections, which returned to control levels when ADAP expression was reconstituted (one-way ANOVA *F*_(2,108)_ = 4.327, *p* < 0.05; *p* < 0.05, control vs. knock down, *p* < 0.05, knock down vs. rescue, *p* < 0.05, Fisher’s PLSD). Axonal structures in contrast were not significantly affected (control 355.06 ± 32.83 knock down 180.3 ± 112.26 rescue 307.3 ± 175.16; *F*_(2,98)_ = 2.321 *p* > 0.05).

### ADAP Knock Down Decreases MAP2 Immunoreactivity in Neurons

**Figure 4:**
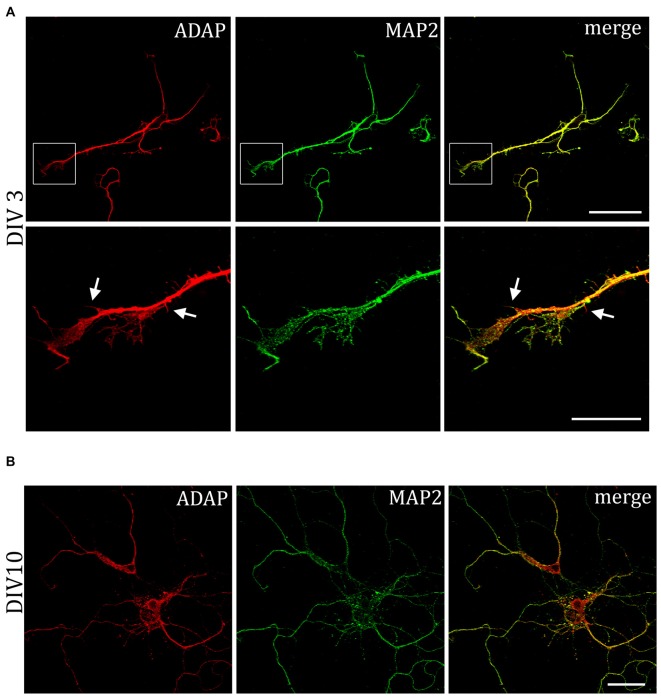
**Association of ADAP with MAP2-positive structures in hippocampal neurons. (A)** Confocal microscopy reveals that during early neurite formation (DIV3), ADAP is richly expressed along outgrowing neurites and highly co-localized with MAP2. Co-localization is evident in both the core neurite and at the growth tip, including filamentous and filopodial structures. Only occasionally, small MAP2-negative filaments appear labeled by ADAP (arrows). Scale bars, 100 μm and 50 μm. **(B)** A high degree of co-localization with MAP2 is also evident at later stages of dendrite development (DIV10). Scale bar, 100 μm.

**Figure 5:**
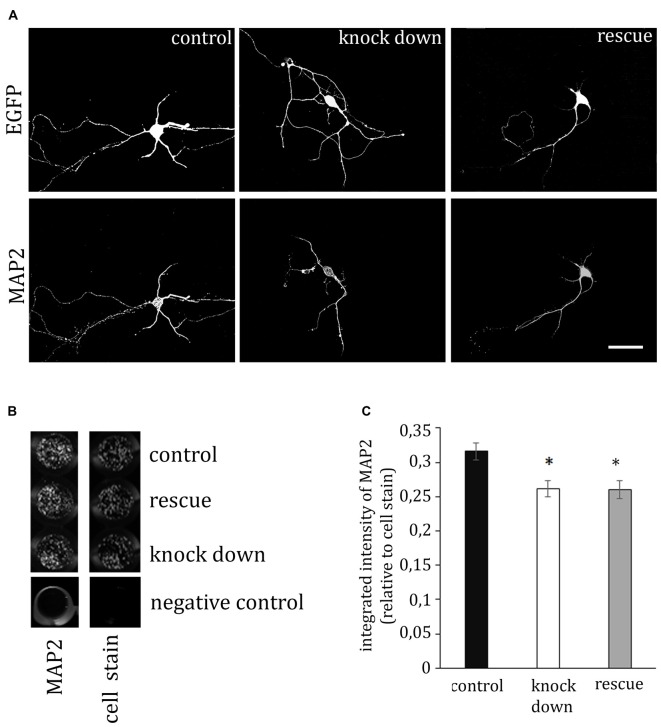
**ADAP stimulates MAP2 expression. (A)** The immunofluorescence signal of the dendritic marker MAP2 is reduced in ADAP knock-down neurons, compared to control transfected neurons. Scale bar, 100 μm. **(B)** MAP2 labeling in transfected primary cultures using the In-Cell-Western method confirmed the overall reduction of MAP2 labeling intensity upon ADAP knock down (*n* = 20–22 wells per condition). Labeling is normalized for the intensity of cell stain, which shows similar density of cells in the different experimental groups. **(C)** Quantification of In-Cell-Westerns confirms a significant reduction of MAP2 labeling intensity in ADAP knock-down samples. In contrast to dendritic growth measurement, co-expression of the rescue construct does not recover MAP2 expression levels. Data are Mean ± SEM. *p < 0.05.

MAP2 is important for the stabilization of microtubules during neurogenesis and is enriched in dendrites, implicating a role in stabilizing dendritic shape during neuron development. When analyzing the morphology of hippocampal neurons, we recognized the close association of ADAP with MAP2-positive structures at different stages of development (Figure 4). Moreover, we noticed an apparent loss of MAP2 immunoreactivity in ADAP-knock-down cells (Figure 5A). To quantify this effect we used the In-Cell Western^TM^ method in acutely transfected neuronal cells. With an average transfection rate of 50% knock down of ADAP significantly decreased MAP2 labeling intensity compared to controls, however, in contrast to integrin labeling and dendrite growth, this effect could not be rescued through ADAP re-expression (Figures 5B,C; *F*_(2,72)_ = 6.034 *p* < 0.005; knock down vs. control *p* < 0.05; rescue vs. control *p* < 0.05).

### ADAP Knock Downs Decreases Baseline NF-κB Activity in Neurons

**Figure 6:**
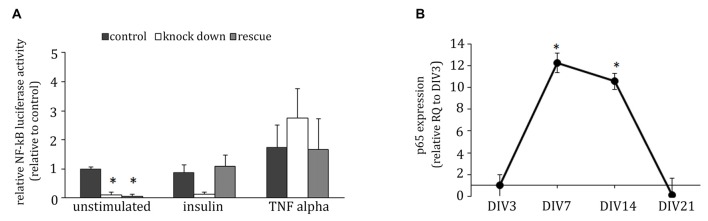
**ADAP reduces baseline NF-κB activity in hippocampal neurons. (A)** Under minimal medium conditions, the NF-κB-induced luciferase signal in primary neurons is reduced in cells with ADAP knock down, irrespective of the presence of the rescue construct. By contrast, when grown under insulin supplementation, a recovery to control levels becomes evident in the rescue group. Application of TNF-α increases luciferase activity in all groups, indicating an efficient activation of the canonical NF-κB pathway (*N* = 3). **(B)** mRNA expression of p65 in primary culture peaks at DIV7 (*N* = 3). Data are Mean ± SEM. *p < 0.05, compared to DIV3.

In T-cells, ADAP is involved in the activation of the transcription factor NF-κB (Medeiros et al., 2007; Srivastava et al., 2010; Thaker et al., 2015). To test for a potential involvement of this ADAP function in neuronal differentiation we examined the activity of NF-κB under conditions of ADAP knock down and reconstitution using a luciferase reporter assay. Indeed, ADAP knock down led to a significant reduction in NF-κB activity under basal differentiation conditions that was not recovered by the rescue construct (*F*_(2,8)_ = 29.558; *p* < 0.001; control vs. knock down *p* < 0.01; control vs. rescue *p* < 0.01; Figure 6A). Under stimulation with insulin, no significant effect of ADAP expression was found (*F*_(2,8)_ = 3.021 *p* > 0.12) but a strong trend for reduction was evident in the knock-down samples. Furthermore, under the stimulation of canonical NF-κB signaling with TNF-α, no difference was observed in NF-κB activity between different ADAP manipulations (*F*_(2,8)_ = 1.432 *p* > 0.31).

The NF-κB family members p65 and c-Rel have previously been described to control neuronal differentiation and plasticity. To determine the potential target of ADAP-mediated NF-κB in neurons, we analyzed the mRNA-expression of p65 and c-Rel in hippocampal primary culture and found a significant p65-mRNA expression with a pronounced increase on DIV7 and DIV14 (one-way ANOVA *F*_(3,32)_ = 3.741 *p* < 0.05; DIV7 vs. DIV21 and DIV14 vs. DIV21 *p* < 0.05; Figure 6B). c-Rel-mRNA in contrast was not detectable in our cultures.

## Discussion

In the current study, we demonstrate the involvement of the hematopoietic scaffold molecule ADAP in dendrite formation of hippocampal neurons. Our data suggest that ADAP in the nervous system may act analogous to ADAP in T-cells, i.e., by assembling a specific signaling complex for the inside-out activation of integrins and by controlling cell differentiation.

Depending on their conformation, integrins display low, intermediate, or high affinity to their ligands. Activation of integrin adhesion can be triggered by inducing an increased proportion of the high-affinity conformations of integrins on the cell surface. Subsequently, ligand binding stimulates integrin clustering (avidity regulation) and association with the actin cytoskeleton to mediate macromolecular adhesion complex formation. Moreover, integrin-ligand binding induces outside-in-signaling to control adhesion, spreading, migration as well as cellular differentiation, survival and proliferation (Abram and Lowell, 2009; Hogg et al., 2011; Margadant et al., 2011).

### ADAP/SKAP-HOM-Module

Research in T-cells has demonstrated that ADAP is a critical factor for the activation of integrins: ADAP and SKAP55, which form a signaling unit “the ADAP/SKAP55-module” to recruit the two Rap1 effector proteins RAPL and RIAM (Rap1–GTP-interacting adapter molecule), the small GTPase Rap1, the Ste20-like kinase MST1, as well as the FERM-domain containing proteins Talin and Kindlin-3 for integrin activation at the plasma membrane (Raab et al., 2011; Kliche et al., 2012; Kasirer-Friede et al., 2014). The formation of these macromolecular complexes is required for integrin activation leading to adhesion and migration and to promote proliferation and differentiation of T-cells (Ménasché et al., 2007; Witte et al., 2012). ADAP and/or SKAP55 (homolog of SKAP-HOM) are crucial for receptor-mediated integrin signaling events in various cell types of the immune system including T-cells, platelets, dendritic cells and neutrophils (Peterson et al., 2001; Griffiths and Penninger, 2002; Togni et al., 2005, 2012; Kasirer-Friede et al., 2007; Wang et al., 2007; Reinhold et al., 2009; Block et al., 2012). Thus ADAP and/or SKAP55 control T-cell adhesion, interactions of T-cells with antigen-presenting cells, and T-cell migration *in vitro* and *in vivo* (Kliche et al., 2006, 2012; Wang et al., 2007, 2009; Burbach et al., 2008; Mitchell et al., 2013). We now demonstrate that ADAP, SKAP-HOM, RAPL, RIAM and MST1 all are expressed in hippocampal tissue and that ADAP, SKAP-HOM, RAPL and MST1 can be co-precipitated under the same conditions as from T-cells. This strongly suggests that an ADAP complex exists in neurons that is comparable to the ADAP/SKAP55/RAPL/MST1 complex in T-cells and might similarly control the activity state of integrins during neuronal development (Warren et al., 2012).

### ADAP is Involved in β1-Integrin Activation

Neurons express various β1- and β3-integrins (Wu and Reddy, 2012) that interact with the rich extracellular matrix of the nervous system (e.g., fibronectin, laminin, or collagen) and diffusible factors that serve as guidance cues mediating migration and neurite growth (e.g., netrins, semaphorins and ephrins; Myers et al., 2011). Overall it seems that in neurons integrins are rather in an open conformation and that further activation occurs by inside-out activation upon excitation or by outside-in mechanism such as high concentration of ligands in the extracellular matrix (Lin et al., 2005; Sekine et al., 2012). In addition, local activation of integrins also directs axon outgrowth or growth cone formation through integrin-recycling (Myers et al., 2011). Gain-of function and loss-of function studies identified several signaling molecules that regulate β1- and β3-integrin function in neurons. These include members of the Arf, Ras and Rho GTPases, integrin-linked kinase, focal adhesion kinase (FAK) and the two FERM-domain containing proteins Talin and Kindlin-1 (Myers et al., 2011; Tan et al., 2012, 2015; Kerstein et al., 2013).

We could show that ADAP and SKAP-HOM are both expressed throughout the soma and dendrites of developing neurons. ADAP could be observed in dendritic growth tips during early development and in association with MAP2-positive dendritic microtubules at different stages of development. This is in line with an association of RAPL and MST1 with microtubules observed in various cell types (Fujita et al., 2005; Oh et al., 2006).

In primary hippocampal culture, β1-integrins are the predominant form in early neuronal development and critical for dendritic differentiation (Schlomann et al., 2009; Warren et al., 2012; Rehberg et al., 2014). Their activation in outgrowing neurites involves a phosphorylation at the cytoplasmic tail, sorting to recycling endosomes and trafficking to the plasma membrane (Schlomann et al., 2009; Tan et al., 2012). Similarly, a re-localization of α5β1 integrins from soma to the dendrite has been demonstrated during dendrite formation and dendritic maturation in neurons of the hippocampus and neocortex (Bi et al., 2001). In T-cells, the ADAP/SKAP55/RAPL/MST1 complex associates with the α-chain of LFA-1 and mediates its intracellular trafficking (Kliche et al., 2012). We now show that suppression of ADAP expression in hippocampal neurons reduces the amount of activated β1-integrin on the surface of outgrowing dendrites, while the labeling for total β1-integrin is increased in this compartment. To be conservative in our evaluation, we did not correct integrin surface labeling for the total size of the cellular compartment. The fact that ADAP knock down induces both reduced 9EG7 labeling and reduction in dendritic growth may thus have led to an underestimation of the former effect. This supports the hypothesis that the ADAP/SKAP-HOM module in neuronal cells may be involved in the inside-out activation of integrins during dendritic differentiation. An apparent redistribution of β1-integrins from the soma to the dendrites of ADAP deficient neurons may occur as a compensatory change in these cells in order to limit the detrimental effects of their reduced “inside-out” activation.

### ADAP Knock Down Reduced MAP2 Expression in Developing Neurons

To assess the effect of ADAP knock down on neuronal differentiation, we analyzed the growth of dendrites and the expression of the differentiation marker MAP2 in these cells. Both indices were significantly reduced upon shRNA-mediated knock down, again, in analogy to the reduced production of differentiation markers in ADAP-deficient T-cells. The reduction of MAP2 levels may be considered as a mere marker of reduced neuronal differentiation in ADAP knock-down cells. However, this phenomenon may also be more directly involved in the process as MAP2 is critical for dendritogenesis and dendritic outgrowth (Bernhard et al., 1985; Harada et al., 2002) and the level of MAP2 expression is controlled by integrin stimulation in developing neurons (Domingo-Espín et al., 2012; Jeon et al., 2012). Axonal growth was not significantly affected in our experiments, but might well be responsive to ADAP manipulation at an earlier time of differentiation.

Various binding partners of ADAP exist that may be involved in integrin activation and dendrite formation of primary neurons. Our data suggest that ADAP may act in association with SKAP-HOM, RAPL/MST1. We did not observe a co-precipitation with the Talin-binding Rap1-effector RIAM, which is found in an independent integrin-activating ADAP/SKAP55 module in T-cells (Kliche et al., 2012). Further, the interaction of ADAP with Ena/VASP (Krause et al., 2000) may control neuritogenesis via reorganization of the actin cytoskeleton differentially in the presence and absence of integrin substrates (Gupton and Gertler, 2010). Several association partners bind ADAP dependent on its phosphorylation through the Src family kinase c-Fyn (Sylvester et al., 2010). c-Fyn, in turn, is involved in integrin activation in mouse hippocampus (Bourgin et al., 2007) and firmly established as a factor of neurite outgrowth including the semaphorin 3A-induced dendritic branching of primary hippocampal neurons (Morita et al., 2006). A possible Fyn-dependent interaction partner of ADAP is Nck2, which has been implicated in growth factor-induced neuritogenesis in PC12 cells (Guan et al., 2007). Moreover, Crk has been specifically implicated in dendritogenesis of hippocampal neurons induced by Reelin, however, it did not affect dendrite growth under unstimulated conditions as employed in our experiments (Matsuki et al., 2008).

### ADAP Deficiency Leads to a Reduction in NF-κB Activity

Finally, reduced neuronal differentiation in ADAP deficient cells may involve the deficits of NF-κB signaling. NF-κB is constitutively active in glutamatergic neurons such as in hippocampus (O’Neill and Kaltschmidt, 1997; Kaltschmidt and Kaltschmidt, 2009) and crucial for dendritic growth, branching and spine number (Gutierrez et al., 2005; Salama-Cohen et al., 2006; O’Sullivan et al., 2010). In T-cells, a pool of ADAP that is not associated to SKAP55 activates the canonical NF-κB pathway after TCR/CD28-stimulation (Medeiros et al., 2007). Our glia-free culture system allowed us to examine cell autonomous ADAP effects in neurons largely independent of stimulation of the NF-κB signaling pathway through glia-derived cytokines. We probed the canonical pathway using exogenous TNFα, but found no deficit in ADAP-deficient cells, although a significantly reduced NF-κB activity was evident under basal conditions. This suggests that ADAP can stimulate NF-κB signaling in developing neurons but may be dispensable for its activation through glia-derived cytokines. Whether the observed decrease of baseline NF-κB activity is due to a reduced integrin-mediated signaling mediated by ADAP/SKAP-HOM/RAPL/MST1 or promoted by ADAP alone remains to be determined. The lack of a rescue in NF-κB activity upon ADAP re-expression might be related to insufficient re-expression levels or a requirement of dynamic rather than constitutive ADAP expression regulation in this function. Nevertheless, the comparison with other knock down effects suggests that ADAP-dependent NF-κB activity is dispensable for dendritic outgrowth, but might play a role in MAP2 expression.

**Figure 7:**
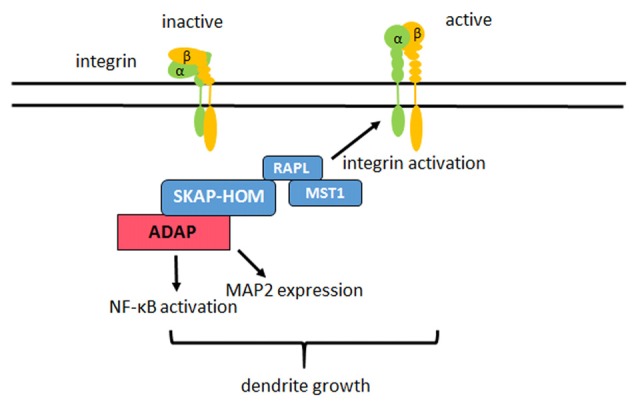
**Model of ADAP actions in neurons.** In hippocampal neurons ADAP exists in a complex with SKAP-HOM, RAPL and MST1. ADAP stimulates the “inside out” activation of β1 integrins on the dendrites of these neurons and enhances dendritic growth in primary hippocampal culture. Modulation of NF-κB activity and MAP2 expression through ADAP may also contribute to the dendritic development, but require further investigation as they could not be rescued through the reconstitution of ADAP expression in our experiments.

In summary, we demonstrate that the hematopoietic adaptor protein ADAP is critical for the “inside-out” activation of β1-integrin and integrin-dependent dendritic differentiation in hippocampal neurons. Several potential interaction partners exist for ADAP that have been implicated in neuronal development, suggesting that this versatile adaptor protein may play a similarly important role as an integrator of intracellular signals during neuronal differentiation as described for T-cell development (Figure 7).

## Author Contributions

MT, JT and IN performed cell culture experiments; SK immunoprecipitation and Western analysis; BM generated ADAP knock-down constructs; MT, SK and OS analyzed data and wrote the manuscript.

## Conflict of Interest Statement

The authors declare that the research was conducted in the absence of any commercial or financial relationships that could be construed as a potential conflict of interest.

## Footnotes

^1^www.brain-maps.org

^2^katahdin.cshl.org

## References

[B1] Abram C. L. Lowell C. A. (2009). The ins and outs of leukocyte integrin signaling. Annu. Rev. Immunol. 27, 339–362. 10.1146/annurev.immunol.021908.132554 19302044 PMC3248397

[B2] Albrecht A. Thiere M. Bergado-Acosta J. R. Poranzke J. Müller B. Stork O. (2013). Circadian modulation of anxiety: a role for somatostatin in the amygdala. PLoS One 8:e84668. 10.1371/journal.pone.0084668 24376834 PMC3869835

[B3] Bernhard R. Huber G. Matus A. (1985). Differences in the developmental patterns of three microtubule-associated proteins in the rat cerebellum. J. Neurosci. 5, 977–991. 3981253 10.1523/JNEUROSCI.05-04-00977.1985 PMC6564990

[B4] Bi X. Lynch G. Zhou J. Gall C. M. (2001). Polarized distribution of α5 integrin in dendrites of hippocampal and cortical neurons. J. Comp. Neurol. 435, 184–193. 10.1002/cne.1201 11391640

[B5] Block H. Herter J. M. Rossaint J. Stadtmann A. Kliche S. Lowell C. A.. (2012). Crucial role of SLP-76 and ADAP for neutrophil recruitment in mouse kidney ischemia-reperfusion injury. J. Exp. Med. 209, 407–421. 10.1084/jem.20111493 22291096 PMC3280874

[B6] Bourgin C. Murai K. K. Richter M. Pasquale E. B. (2007). The EphA4 receptor regulates dendritic spine remodeling by affecting β1-integrin signaling pathways. J. Cell Biol. 178, 1295–1307. 10.1083/jcb.200610139 17875741 PMC2064660

[B7] Burbach B. J. Srivastava R. Ingram M. A. Mitchell J. S. Shimizu Y. (2011). The pleckstrin homology domain in the SKAP55 adapter protein defines the ability of the adapter protein ADAP to regulate integrin function and NF-kappaB activation. J. Immunol. 186, 6227–6237. 10.4049/jimmunol.1002950 21525391 PMC3108501

[B8] Burbach B. J. Srivastava R. Medeiros R. B. Gorman W. E. O. Peterson E. J. Shimizu Y. (2008). Distinct regulation of integrin-dependent T-cell conjugate. J. Immunol. 181, 4840–4851. 10.4049/jimmunol.181.7.4840 18802088 PMC2593878

[B9] Dent E. W. Gupton S. L. Gertler F. B. (2011). The growth cone cytoskeleton in axon outgrowth and guidance. Cold Spring Harb. Perspect. Biol. 3:a001800. 10.1101/cshperspect.a001800 21106647 PMC3039926

[B10] Domingo-Espín J. Petegnief V. de Vera N. Conchillo-Solé O. Saccardo P. Unzueta U.. (2012). RGD-based cell ligands for cell-targeted drug delivery act as potent trophic factors. Nanomedicine 8, 1263–1266. 10.1016/j.nano.2012.06.005 22841914

[B11] Engelmann S. Togni M. Kliche S. Reinhold D. Schraven B. Reinhold A. (2015). The adhesion- and degranulation-promoting adaptor protein and its role in the modulation of experimental autoimmune encephalomyelitis. Crit. Rev. Immunol. 35, 1–14. 10.1615/critrevimmunol.2014012162 25746044

[B12] Fujita H. Fukuhara S. Sakurai A. Yamagishi A. Kamioka Y. Nakaoka Y.. (2005). Local activation of Rap1 contributes to directional vascular endothelial cell migration accompanied by extension of microtubules on which RAPL, a Rap1-associating molecule, localizes. J. Biol. Chem. 280, 5022–5031. 10.1074/jbc.m409701200 15569673

[B13] Griffiths E. K. Penninger J. M. (2002). ADAP-ting TCR signaling to integrins. Sci. STKE 2002:re3. 10.1126/stke.2002.127.re3 11943877

[B14] Guan S. Chen M. Woodley D. Li W. (2007). Nckβ adapter controls neuritogenesis by maintaining the cellular paxillin level. Mol. Cell. Biol. 27, 6001–6011. 10.1128/mcb.01807-06 17591694 PMC1952161

[B15] Gupton S. L. Gertler F. B. (2010). Integrin signaling switches the cytoskeletal and exocytic machinery that drives neuritogenesis. Dev. Cell 18, 725–736. 10.1016/j.devcel.2010.02.017 20493807 PMC3383070

[B16] Gutierrez H. Hale V. A. Dolcet X. Davies A. (2005). NF-κB signalling regulates the growth of neural processes in the developing PNS and CNS. Development 132, 1713–1726. 10.1242/dev.01702 15743881

[B17] Harada A. Teng J. Takei Y. Oguchi K. Hirokawa N. (2002). MAP2 is required for dendrite elongation, PKA anchoring in dendrites and proper PKA signal transduction. J. Cell Biol. 158, 541–549. 10.1083/jcb.200110134 12163474 PMC2173814

[B18] Hogg N. Patzak I. Willenbrock F. (2011). The insider’s guide to leukocyte integrin signalling and function. Nat. Rev. Immunol. 11, 416–426. 10.1038/nri2986 21597477

[B19] Horn J. Wang X. Reichardt P. Stradal T. E. Warnecke N. Simeoni L.. (2009). Src homology 2-domain containing leukocyte-specific phosphoprotein of 76 kDa is mandatory for TCR-mediated inside-out signaling, but dispensable for CXCR4-mediated LFA-1 activation, adhesion and migration of T cells. J. Immunol. 183, 5756–5767. 10.4049/jimmunol.0900649 19812192

[B22] Jeon W. B. Park B. H. Choi S. K. Lee K.-M. Park J.-K. (2012). Functional enhancement of neuronal cell behaviors and differentiation by elastin-mimetic recombinant protein presenting Arg-Gly-Asp peptides. BMC Biotechnol. 12:61. 10.1186/1472-6750-12-61 22978264 PMC3507755

[B23] Kaech S. Banker G. (2006). Culturing hippocampal neurons. Nat. Protoc. 1, 2406–2415. 10.1038/nprot.2006.356 17406484

[B24] Kaltschmidt B. Kaltschmidt C. (2009). NF-κB in the nervous system. Cold Spring Harb. Perspect. Biol. 1:a001271. 10.1101/cshperspect.a001271 20066105 PMC2773634

[B25] Kasirer-Friede A. Kang J. Kahner B. Ye F. Ginsberg M. H. Shattil S. J. (2014). ADAP interactions with talin and kindlin promote platelet integrin αIIβ3 activation and stable fibrinogen binding. Blood 123, 3156–3165. 10.1182/blood-2013-08-520627 24523237 PMC4023421

[B26] Kasirer-Friede A. Moran B. Nagrampa-Orje J. Swanson K. Ruggeri Z. M. Schraven B.. (2007). ADAP is required for normal alphaIIbeta3 activation by VWF/GP Ib-IX-V and other agonists. Blood 109, 1018–1025. 10.1182/blood-2006-05-022301 17003372 PMC1785130

[B27] Kerstein P. C. Jacques-Fricke B. T. Rengifo J. Mogen B. J. Williams J. C. Gottlieb P. A.. (2013). Mechanosensitive TRPC1 channels promote calpain proteolysis of talin to regulate spinal axon outgrowth. J. Neurosci. 33, 273–285. 10.1523/JNEUROSCI.2142-12.2013 23283340 PMC3539200

[B28] Kliche S. Breitling D. Togni M. Pusch R. Heuer K. Wang X.. (2006). The ADAP/SKAP55 signaling module regulates T-cell receptor-mediated integrin activation through plasma membrane targeting of Rap1. Mol. Cell. Biol. 26, 7130–7144. 10.1128/mcb.00331-06 16980616 PMC1592884

[B29] Kliche S. Worbs T. Wang X. Degen J. Patzak I. Meineke B.. (2012). CCR7-mediated LFA-1 functions in T cells are regulated by 2 independent ADAP/SKAP55 modules. Blood 119, 777–785. 10.1182/blood-2011-06-362269 22117043

[B30] Krause M. Sechi A. S. Konradt M. Monner D. Gertler F. B. Wehland J. (2000). Fyn-binding protein (Fyb)/SLP-76-associated protein (SLAP), Ena/vasodilator-stimulated phosphoprotein (VASP) proteins and the Arp2/3 complex link T cell receptor (TCR) signaling to the actin cytoskeleton. J. Cell Biol. 149, 181–194. 10.1083/jcb.149.1.181 10747096 PMC2175102

[B31] Lin C.-Y. Lynch G. Gall C. M. (2005). AMPA receptor stimulation increases alpha5beta1 integrin surface expression, adhesive function and signaling. J. Neurochem. 94, 531–546. 10.1111/j.1471-4159.2005.03203.x 16000124 PMC2366053

[B33] Margadant C. Monsuur H. N. Norman J. C. Sonnenberg A. (2011). Mechanisms of integrin activation and trafficking. Curr. Opin. Cell Biol. 23, 607–614. 10.1016/j.ceb.2011.08.005 21924601

[B34] Marie-Cardine A. Verhagen A. M. Eckerskorn C. Schraven B. (1998). SKAP-HOM, a novel adaptor protein homologous to the FYN-associated protein SKAP55. FEBS Lett. 435, 55–60. 10.1016/s0014-5793(98)01040-0 9755858

[B35] Marrs G. S. Honda T. Fuller L. Thangavel R. Balsamo J. Lilien J.. (2006). Dendritic arbors of developing retinal ganglion cells are stabilized by β1-integrins. Mol. Cell. Neurosci. 32, 230–241. 10.1016/j.mcn.2006.04.005 16757177

[B36] Matsuki T. Pramatarova A. Howell B. W. (2008). Reduction of Crk and CrkL expression blocks reelin-induced dendritogenesis. J. Cell Sci. 121, 1869–1875. 10.1242/jcs.027334 18477607 PMC2430739

[B37] Medeiros R. B. Burbach B. J. Mueller K. L. Srivastava R. Moon J. J. Highfill S.. (2007). Regulation of NF-κB activation in T-cells via association of the adapter proteins ADAP and CARMA1. Science 316, 754–758. 10.1126/science.1137895 17478723

[B38] Ménasché G. Kliche S. Bezman N. Schraven B. (2007). Regulation of T-cell antigen receptor-mediated inside-out signaling by cytosolic adapter proteins and Rap1 effector molecules. Immunol. Rev. 218, 82–91. 10.1111/j.1600-065x.2007.00543.x 17624945

[B39] Mikenberg I. Widera D. Kaus A. Kaltschmidt B. Kaltschmidt C. (2007). Transcription factor NF-κB is transported to the nucleus via cytoplasmic dynein/dynactin motor complex in hippocampal neurons. PLoS One 2:e589. 10.1371/journal.pone.0000589 17622342 PMC1899224

[B40] Mitchell J. S. Burbach B. J. Srivastava R. Fife B. T. Shimizu Y. (2013). Multistage T cell—dendritic cell interactions control optimal CD4 T cell activation through the ADAP-SKAP55-signaling module. J. Immunol. 191, 2372–2383. 10.4049/jimmunol.1300107 23918975 PMC3772631

[B41] Moresco E. M. Y. Donaldson S. Williamson A. Koleske A. J. (2005). Integrin-mediated dendrite branch maintenance requires abelson (Abl) family kinases. J. Neurosci. 25, 6105–6118. 10.1523/JNEUROSCI.1432-05.2005 15987940 PMC6725048

[B42] Morita A. Yamashita N. Sasaki Y. Uchida Y. Nakajima O. Nakamura F.. (2006). Regulation of dendritic branching and spine maturation by semaphorin3A-fyn signaling. J. Neurosci. 26, 2971–2980. 10.1523/JNEUROSCI.5453-05.2006 16540575 PMC6673984

[B43] Musci M. A. Hendricks-Taylor L. R. Motto D. G. Paskind M. Kamens J. Turck C. W.. (1997). Molecular cloning of SLAP-130, an SLP-76-associated substrate of the T cell antigen receptor-stimulated protein tyrosine kinases. J. Biol. Chem. 272, 11674–11677. 10.1074/jbc.272.18.11674 9115214

[B44] Myers J. P. Santiago-Medina M. Gomez T. M. (2011). Regulation of axonal outgrowth and pathfinding by integrin-ECM interactions. Dev. Neurobiol. 71, 901–923. 10.1002/dneu.20931 21714101 PMC3192254

[B45] O’Neill L. A. Kaltschmidt C. (1997). NF-κ B: a crucial transcription factor for glial and neuronal cell function. Trends Neurosci. 20, 252–258. 10.1016/s0166-2236(96)01035-1 9185306

[B46] Oh H. J. Lee K. K. Song S. J. Jin M. S. Song M. S. Lee J. H.. (2006). Role of the tumor suppressor RASSF1A in Mst1-mediated apoptosis. Cancer Res. 66, 2562–2569. 10.1158/0008-5472.CAN-05-2951 16510573

[B607] O’Sullivan N. C. Croydon L. McGettigan P. A. Pickering M. Murphy K. J. (2010). Hippocampal region-specific regulation of NF-κB may contribute to learning-associated synaptic reorganisation. Brain Res. Bull. 81, 385–390. 10.1016/j.brainresbull.2009.11.001 19909798

[B47] Peterson E. J. (2003). The TCR ADAPts to integrin-mediated cell adhesion. Immunol. Rev. 192, 113–121. 10.1034/j.1600-065x.2003.00026.x 12670399

[B48] Peterson E. J. Woods M. L. Dmowski S. A. Derimanov G. Jordan M. S. Wu J. N.. (2001). Coupling of the TCR to integrin activation by Slap-130/Fyb. Science 293, 2263–2265. 10.1126/science.1063486 11567141

[B49] Raab M. Smith X. Matthess Y. Strebhardt K. Rudd C. E. (2011). SKAP1 protein PH domain determines RapL membrane localization and Rap1 protein complex formation for T cell receptor (TCR) activation of LFA-1. J. Biol. Chem. 286, 29663–29670. 10.1074/jbc.M111.222661 21669874 PMC3191007

[B50] Rehberg K. Kliche S. Madencioglu D. A. Thiere M. Müller B. Meineke B. M.. (2014). The serine/threonine kinase ndr2 controls integrin trafficking and integrin-dependent neurite growth. J. Neurosci. 34, 5342–5354. 10.1523/JNEUROSCI.2728-13.2014 24719112 PMC6609001

[B51] Reinhold A. Reimann S. Reinhold D. Schraven B. Togni M. (2009). Expression of SKAP-HOM in DCs is required for an optimal immune response *in vivo*. J. Leukoc. Biol. 86, 61–71. 10.1189/jlb.0608344 19369640

[B52] Rubinson D. A. Dillon C. P. Kwiatkowski A. V. Sievers C. Yang L. Kopinja J.. (2003). A lentivirus-based system to functionally silence genes in primary mammalian cells, stem cells and transgenic mice by RNA interference. Nat. Genet. 33, 401–406. 10.1038/ng1117 12590264

[B53] Salama-Cohen P. Arévalo M.-Á. Grantyn R. Rodríguez-Tébar A. (2006). Notch and NGF/p75NTR control dendrite morphology and the balance of excitatory/inhibitory synaptic input to hippocampal neurones through Neurogenin 3. J. Neurochem. 97, 1269–1278. 10.1111/j.1471-4159.2006.03783.x 16539652

[B54] Schlomann U. Schwamborn J. C. Müller M. Fässler R. Püschel A. W. (2009). The stimulation of dendrite growth by Sema3A requires integrin engagement and focal adhesion kinase. J. Cell Sci. 122, 2034–2042. 10.1242/jcs.038232 19454481

[B55] Schmid R. S. Anton E. S. (2003). Role of integrins in the development of the cerebral cortex. Cereb. Cortex 13, 219–224. 10.1093/cercor/13.3.219 12571112

[B56] Schultz C. König H.-G. Del Turco D. Politi C. Eckert G. P. Ghebremedhin E.. (2006). Coincident enrichment of phosphorylated IκBα, activated IKK and phosphorylated p65 in the axon initial segment of neurons. Mol. Cell. Neurosci. 33, 68–80. 10.1016/j.mcn.2006.06.008 16875840

[B57] Sekine K. Kawauchi T. Kubo K.-I. Honda T. Herz J. Hattori M.. (2012). Reelin controls neuronal positioning by promoting cell-matrix adhesion via inside-out activation of integrin α5β1. Neuron 76, 353–369. 10.1016/j.neuron.2012.07.020 23083738 PMC3479437

[B58] Sholl D. A. (1953). Dendritic organization in the neurons of the visual and motor cortices of the cat. J. Anat. 87, 387–406. 13117757 PMC1244622

[B59] Srivastava R. Burbach B. J. Shimizu Y. (2010). NF-κB activation in T cells requires discrete control of IκB kinase α/β (IKKalpha/beta) phosphorylation and IKKγ ubiquitination by the ADAP adapter protein. J. Biol. Chem. 285, 11100–11105. 10.1074/jbc.m109.068999 20164171 PMC2856986

[B60] Sylvester M. Kliche S. Lange S. Geithner S. Klemm C. Schlosser A.. (2010). Adhesion and degranulation promoting adapter protein (ADAP) is a central hub for phosphotyrosine-mediated interactions in T cells. PLoS One 5:e11708. 10.1371/journal.pone.0011708 20661443 PMC2908683

[B61] Tan C. L. Andrews M. R. Kwok J. C. F. Heintz T. G. P. Gumy L. F. Fässler R.. (2012). Kindlin-1 enhances axon growth on inhibitory chondroitin sulfate proteoglycans and promotes sensory axon regeneration. J. Neurosci. 32, 7325–7335. 10.1523/JNEUROSCI.5472-11.2012 22623678 PMC6622300

[B62] Tan C. L. Kwok J. C. F. Heller J. P. D. Zhao R. Eva R. Fawcett J. W. (2015). Full length talin stimulates integrin activation and axon regeneration. Mol. Cell. Neurosci. 68, 1–8. 10.1016/j.mcn.2015.03.011 25771432 PMC4604251

[B63] Thaker Y. R. Schneider H. Rudd C. E. (2015). TCR and CD28 activate the transcription factor NF-κB in T-cells via distinct adaptor signaling complexes. Immunol. Lett. 163, 113–119. 10.1016/j.imlet.2014.10.020 25455592 PMC4286576

[B64] Togni M. Engelmann S. Reinhold D. Schraven B. Reinhold A. (2012). The adapter protein ADAP is required for selected dendritic cell functions. Cell Commun. Signal. 10:14. 10.1186/1478-811x-10-14 22672517 PMC3403907

[B65] Togni M. Swanson K. D. Reimann S. Kliche S. Pearce A. C. Simeoni L.. (2005). Regulation of *in vitro* and *in vivo* immune functions by the cytosolic adaptor protein SKAP-HOM. Mol. Cell. Biol. 25, 8052–8063. 10.1128/MCB.25.18.8052-8063.2005 16135797 PMC1234325

[B67] Wang H. Liu H. Lu Y. Lovatt M. Wei B. Rudd C. E. (2007). Functional defects of SKAP-55-deficient T cells identify a regulatory role for the adaptor in LFA-1 adhesion. Mol. Cell. Biol. 27, 6863–6875. 10.1128/MCB.00556-07 17646386 PMC2099233

[B66] Wang H. Rudd C. E. (2008). SKAP-55, SKAP-55-related and ADAP adaptors modulate integrin-mediated immune-cell adhesion. Trends Cell Biol. 18, 486–493. 10.1016/j.tcb.2008.07.005 18760924 PMC3512129

[B68] Wang H. Wei B. Bismuth G. Rudd C. E. (2009). SLP-76-ADAP adaptor module regulates LFA-1 mediated costimulation and T cell motility. Proc. Natl. Acad. Sci. U S A 106, 12436–12441. 10.1073/pnas.0900510106 19617540 PMC2710989

[B69] Warren M. S. Bradley W. D. Gourley S. L. Lin Y.-C. Simpson M. A. Reichardt L. F.. (2012). Integrin β1 signals through Arg to regulate postnatal dendritic arborization, synapse density and behavior. J. Neurosci. 32, 2824–2834. 10.1523/JNEUROSCI.3942-11.2012 22357865 PMC3313657

[B70] Witte A. Degen J. Baumgart K. Waldt N. Kuropka B. (2012). Emerging roles of ADAP, SKAP55 and SKAP-HOM for integrin and NF-κB signaling in T cells. J. Clin. Cell. Immunol. S12:002 10.4172/2155-9899.S12-002

[B71] Wu X. Reddy D. S. (2012). Integrins as receptor targets for neurological disorders. Pharmacol. Ther. 134, 68–81. 10.1016/j.pharmthera.2011.12.008 22233753 PMC3288359

[B72] Xiang H. Hochman D. W. Saya H. Fujiwara T. Schwartzkroin P. A. Morrison R. S. (1996). Evidence for p53-mediated modulation of neuronal viability. J. Neurosci. 16, 6753–6765. 8824316 10.1523/JNEUROSCI.16-21-06753.1996 PMC6579268

[B73] Yang D. Bruun D. Andreas D. Lein P. (2010). Method for shipping live cultures of dissociated rat hippocampal neurons. Curr. Neurobiol. 1, 95–98. 24052689 PMC3775285

[B74] Zhang Y. Wang H. (2012). Integrin signalling and function in immune cells. Immunology 135, 268–275. 10.1111/j.1365-2567.2011.03549.x 22211918 PMC3372743

